# Supplementary material for: Generative design of synthetic gene circuits for functional and evolutionary properties
Source: NPJ Syst Biol Appl. 2026 Mar 16;12:62. doi: 10.1038/s41540-026-00683-6 (PMC13136488; doi:10.1038/s41540-026-00683-6)
Supplement: Supplementary file 1 — Supplementary information [file 41540_2026_683_MOESM1_ESM.pdf]

Supplementary information: Generative design of synthetic gene circuits for functional and evolutionary properties

Olivia Gallup[1] and Harrison Steel[1]

[1]Department of Engineering Science, University of Oxford, Oxford, UK

January 21, 2026

Note 1: Adaptation motifs

More intuitively, the buffer and proportioner motifs differ mainly in terms of how directly the signal is relayed to the output and how the output regains its original state. The buffer motif can be enacted in several ways, for example when the output node 3 only interacts with auxiliary node 2 and with itself (k_{23} and k_{33}) and relies on node 2 to buffer the signal from input node 1 (i.e., soak up and slowly release the signal so that the output reacts prior to steadying out again). The buffer mechanism can also be enacted by output node 3 if the correct balance between its interactions with node 1 and with itself is struck (k_{13} and k_{33}), which is more stable if node 2 is self-binding to interfere less (since some background binding exists even when interaction energy is effectively zero). The proportioner motif gets activated in proportion to the signal and exerts an opposite force on the output, which can be enacted by node 2 if it is not interacting with node 3 directly and still soaks up some of the signal. We thus also expect that the model will identify interactions with node 2 as the determining factor for motifs within adaptation-capable circuits.

Note 2: Adaptable circuits with different ruggedness

The topologies of the two circuits are quite similar – both have a strong output node 3 self-interaction (k_{33}) and an interaction between RNA nodes 2 and 3 (k_{23}). However, the more rugged circuit has a strong interaction between the input and output nodes (k_{13}) and relies more on the interaction between the output and auxiliary nodes (k_{23}) to enable adaptation. Meanwhile, the lower ruggedness circuit has a more stable indirect buffer motif thanks to its interaction between the input and the auxiliary nodes (k_{12}) that distributes the adaptation mechanism across k_{12} and k_{23}. This also reverses the direction of the signal response, as the signal RNA node 1 first binds with RNA node 2 and briefly frees up the output RNA node 3.

Note 3: Motif clusters differing by ruggedness

Some of the motif groups are quite similar. For example, the stable motifs 2 and 3 differ in the strength of their node 2 self-interaction k_{22}, which is stronger than the k_{33} interaction in motif 2 but similar or weaker in motif 3. Otherwise, both utilise self-interactions on all nodes to enact the adaptable mechanism, along with a k_{13} interaction (as we have seen in Figure 2). The self-interaction on node 2 is essentially strengthening the stability of the adaptation mechanism enacted between nodes 1 and 3 by keeping node 2 out of the way from interfering. In comparison, k_{22} is lacking in motif 5 and subsequently has a mix of stable and evolvable circuits. Motif 4 lacks yet another self-interaction by substituting the k_{11} interaction with a k_{23}

interaction, meaning that node 2 acts to stabilise the output node 3 directly. The k_{13} interaction in this motif cluster also only exists in quite a narrow range of bonding energies, which may explain why a substantial proportion of circuits from this cluster display high ruggedness. Finally, motif 1 takes this even further and has no self-interactions, enacting adaptation through k_{12} and k_{13} being weaker than k_{23}. This is the most unstable motif and can be rendered non-functional through perturbations in any interactions, while more stable motifs like 2 and 3 can afford mutations to some of their interactions (e.g. k_{22}) without losing adaptation.

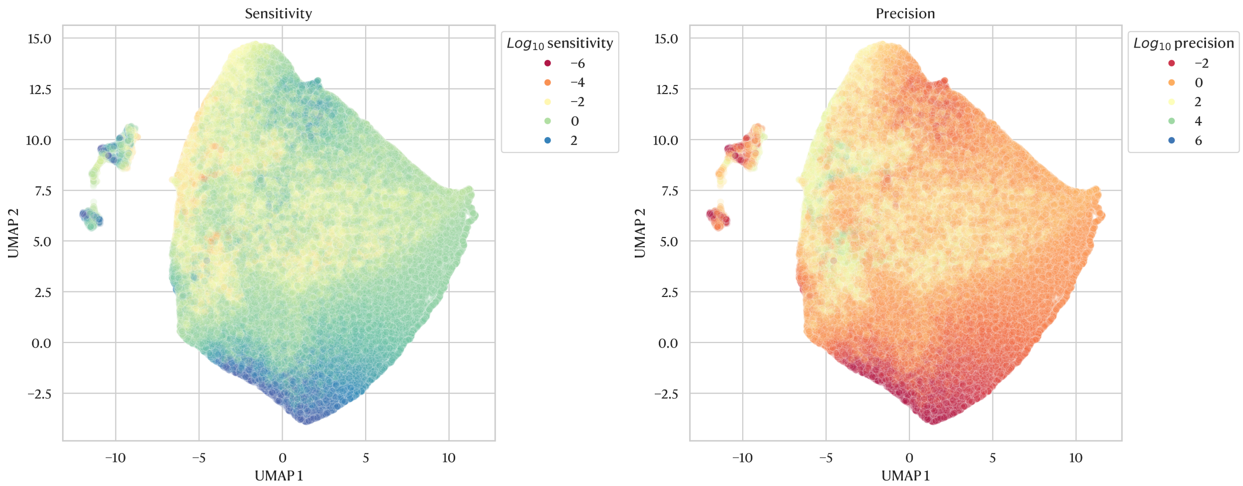

Supplementary Figure 1: UMAP dimensionality reduction applied directly to the simulated RNA circuit topologies produces a latent space that reveals few features. Multiple regions correspond to high or low circuit functionality for both sensitivity and precision. While some trends do emerge just from features in the data, these are structures and rather grouped into one major cluster, with 2-3 very small satellite clusters. This warrants the use of a more sophisticated model like the CVAE to produce improved embeddings.

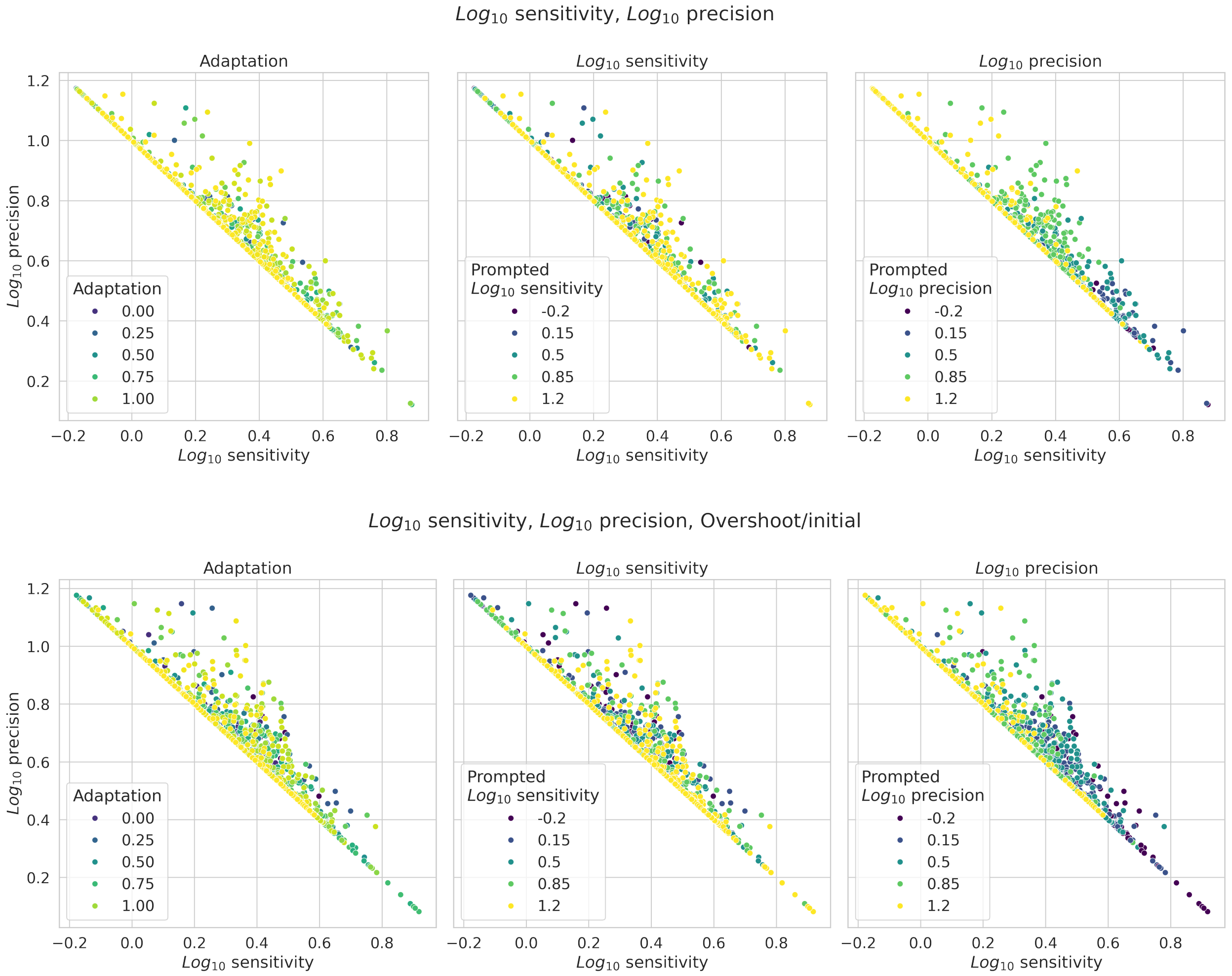

Supplementary Figure 2: Sensitivity vs. precision of gene circuits generated by the CVAE when trained with different objective functions, all alternative ways to express adaptation. For testing different objectives, some of the prompt distributions in Figure 3 were in the wrong order compared to the prompt that generated them. In the sensitivity vs. precision plots above, the prompt colours the samples, with the log_{10} sensitivity and log_{10} precision prompts being taken directly from the prompt and the adaptation prompt having been calculated from those two. For the top row, the model was trained with the objective [log_{10} sensitivity, log_{10} precision] and shows poor adherence to the adaptation and sensitivity prompts, but lines up much better with precision. The same can be said for the bottom row, where the model was trained with the objective [log_{10} sensitivity, log_{10} precision, overshoot/initial], though this one corresponds noticeably better to the sensitivity prompt. The precision portion of the prompt is still dominating the overall prompt. This could be because precision is achieved more simply compared to sensitivity and because sensitivity is the inverse of precision when there is no overshoot.

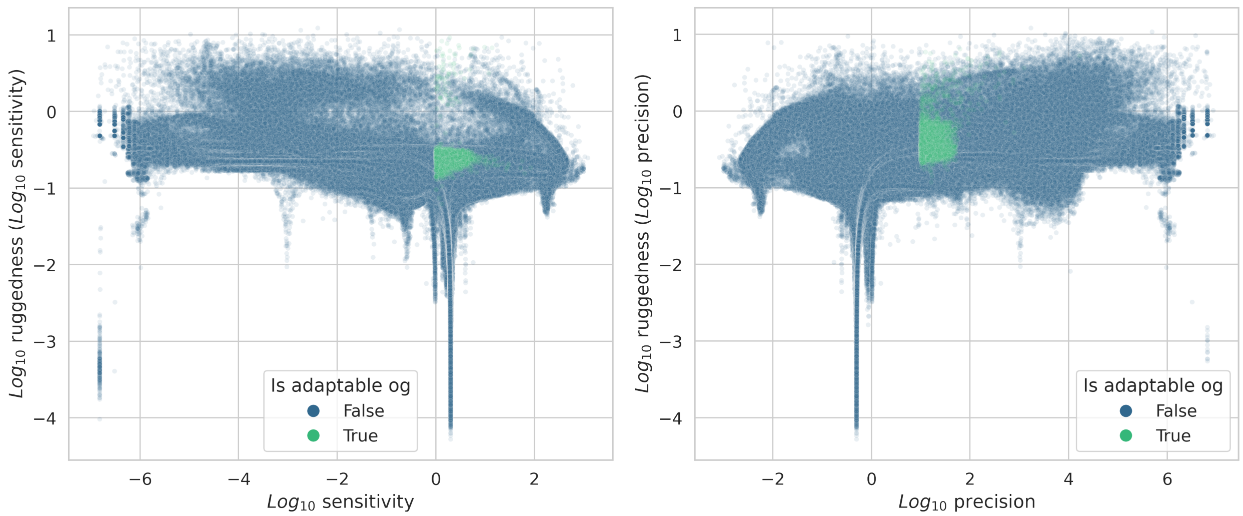

Supplementary Figure 3: Evolutionary ruggedness of sensitivity and precision highlighted by adaptability. Plotting the ruggedness of sensitivity and precision offers a point of reference when examining the ruggedness of adaptation vs. actual adaptation. Both sensitivity and precision plotted against their ruggedness show a mostly flat correlation, with some exceptions for circuits with particularly low ruggedness near sensitivity $= 1$ and precision $= 1$. A subset of circuits with very low sensitivity and high precision also have slightly increased ruggedness. The adaptable region is shown in green as the original adaptable region, meaning the binary threshold definition of sensitivity > 10 and precision > 1 as opposed to a threshold in the custom adaptation function.

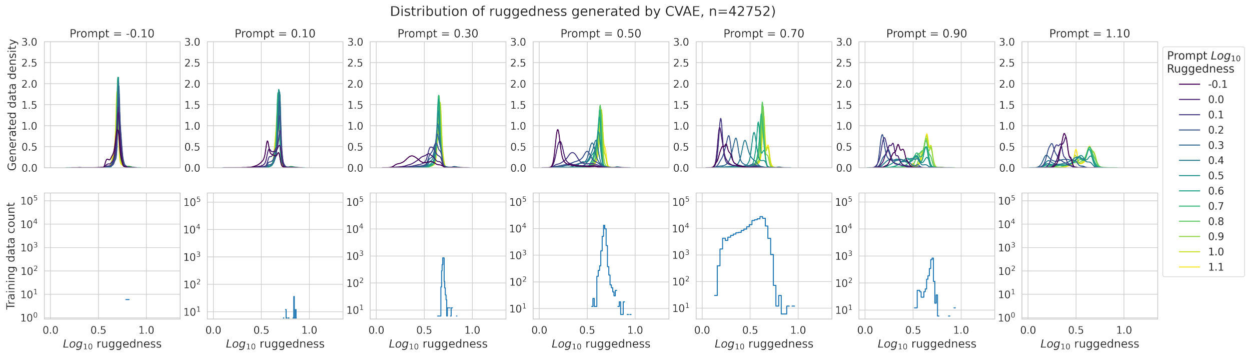

Supplementary Figure 4: The training data ruggedness is not evenly distributed across all levels of adaptation. In Figure 4, we showed the ruggedness prompt distributions for just the highest adaptation prompt. Here, we should the conditional distributions across 7 increasing adaptation ranges, with each distribution representing the true ruggedness of circuits generated for a ruggedness prompt. The adaptation prompt 0.70 is also the adaptation level around which training data is most plentiful.

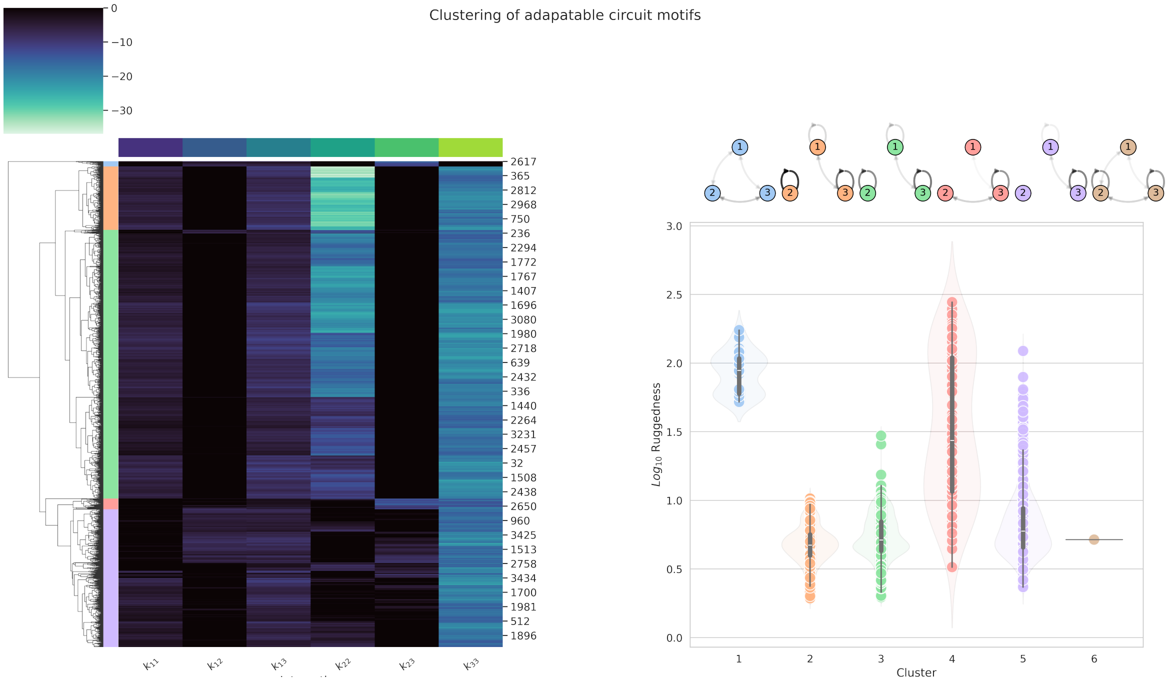

Supplementary Figure 5: Hierarchical clustering of the binding energy interactions identifies 6 motif groups for adaptable circuits generated by the CVAE trained with adaptation and evolutionary ruggedness. One of these clusters (”6”) is only made up of one circuit, so we dropped this circuit in subsequent analyses. The hierarchical clustering plot is shown on the left, with colour blocks on the y-axis corresponding to the same coloured circuits in the scatter-violin plot on the right.

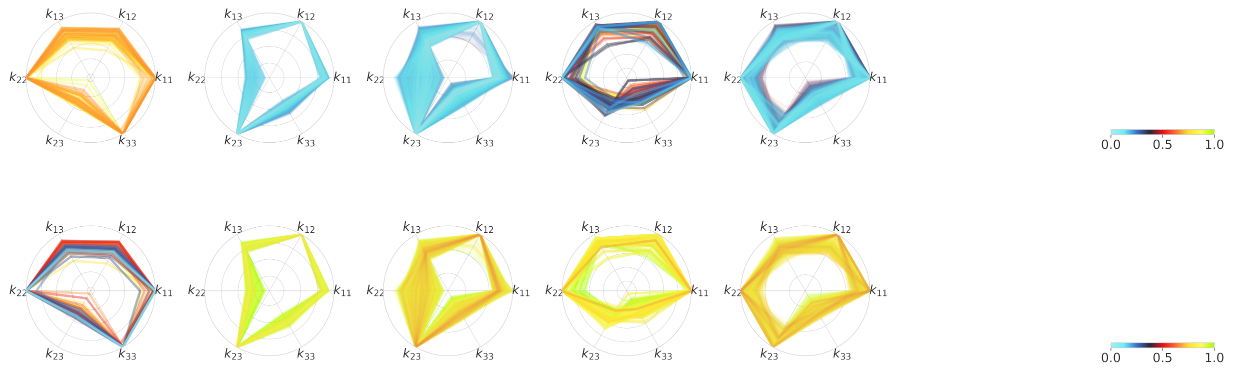

Supplementary Figure 6: Visualisation of the circuit topologies and their ruggedness, grouped by motif. Radar plots of the 5 adaptable motif groups (identified in Figure 4 and Supplementary Figure 5) with different evolutionary ruggedness show the connectivity between different interaction strengths and how these circuit constellations correspond to evolvability. The outside of each radar circle represents a binding interaction (minimum free energy) of 0, while the centre of the circle represents the maximum (approximately 35 kcal/mol), though we are only aiming to show relative differences here. In the top row, circuit motifs are coloured by the log_{10} ruggedness, normalised to the range [0, 1]. The bottom row shows the same motifs coloured by the ruggedness prompt that generated the circuit. Because of the normalisation, the exact values do not line up in terms of hue, but we are only interested in whether the CVAE identifies motifs that are more on the borderline of being evolutionarily stable. In the first motif for example, the circuits closer to the inside of the circle are more rugged, which is in line with the relative prompts of the CVAE. However, the CVAE also overestimated the region of stability for this motif. Something similar can be said for the fourth and fifth motifs, which also have less stable circuits on the inner edges of all motifs that are reflected by the prompt. Overall, the CVAE over-estimates the relative evolutionary stability of circuits, while prompts that are intended to be rugged also result in more rugged circuits.

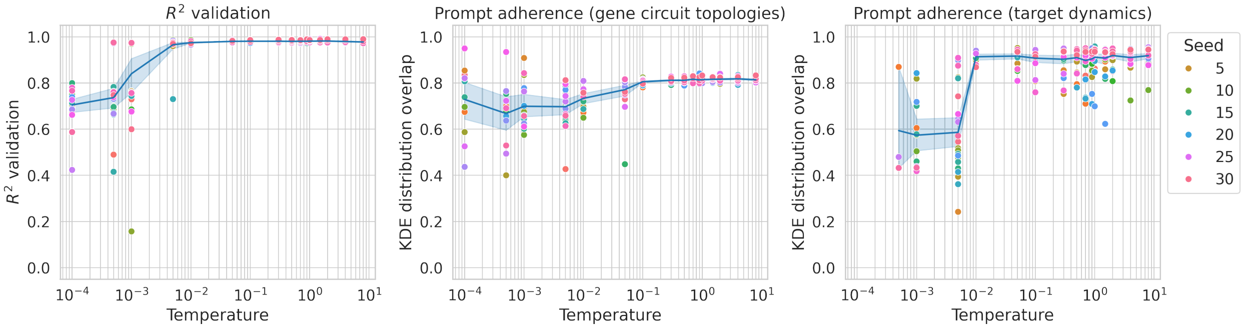

Supplementary Figure 7: Assessment of the contrastive loss. The temperature setting controls how strongly contrastive loss is applied, with a lower temperature punishing incorrect similarities between samples more. The training accuracy (measured by R^2) decreases with temperatures $< 10^{-2}$, so this is the minimum bound. Prompt adherence is assessed by how distinct the distributions of circuits are for our prompts, which is measured by the average overlap between a KDE prompt distributions and all other prompt distributions, averaged over all prompts. The distributions can be in terms of the circuit topologies, so how similar each unique interaction is distributed for each prompt, and in terms of the circuit's function, which has to be simulated once circuits are generated. While there is mild improvement in prompt adherence based on circuit topologies (e.g. a lower average KDE distribution overlap), the same is not true for the actual simulated circuit function of adaptation. In fact, higher temperature (weaker contrastive loss) actually results in some models with decreasing KDE distribution overlaps for adaptation, meaning that these models produce more distinct distributions for each prompt.

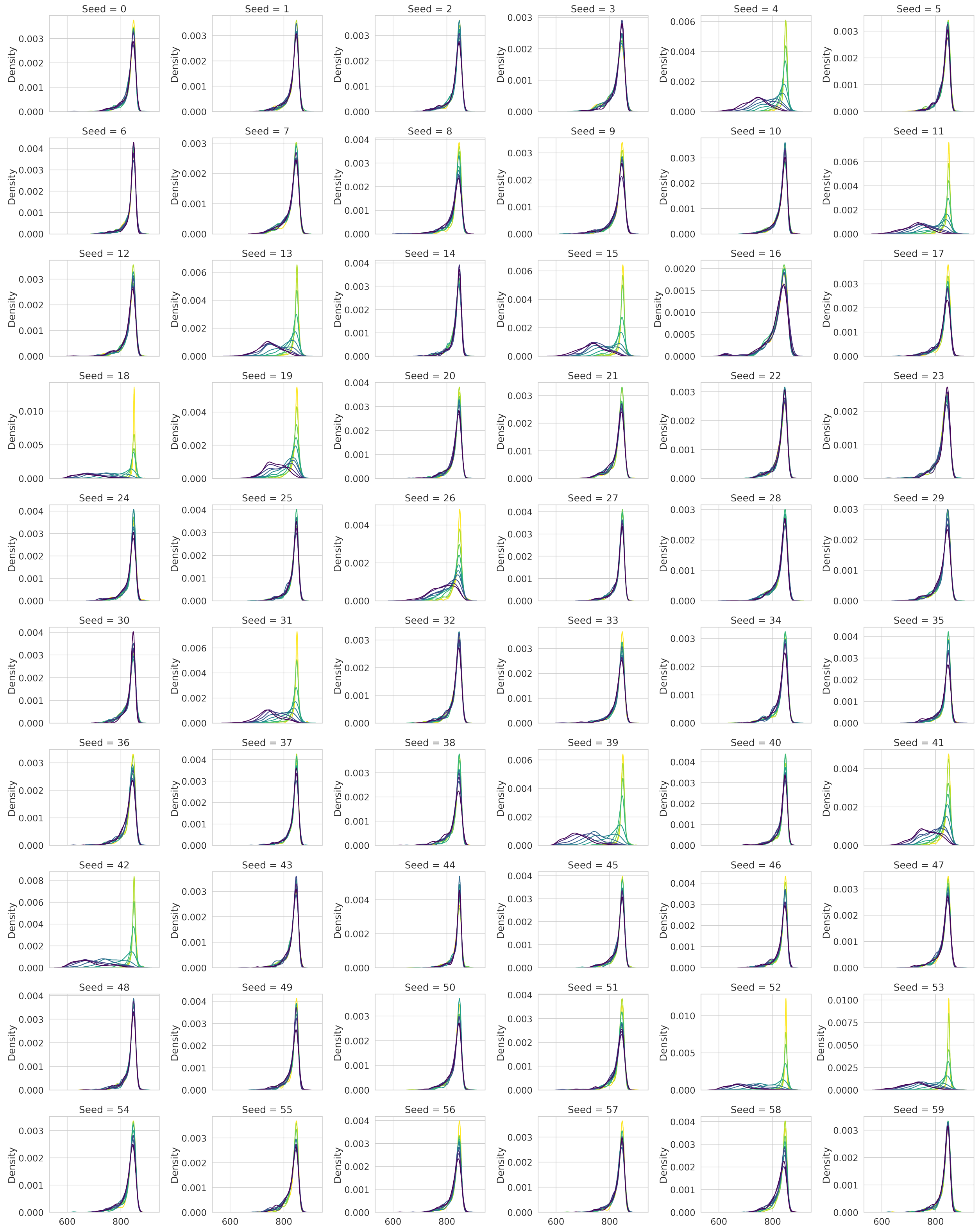

Supplementary Figure 8: Posterior collapse in CVAE based on random seed. 60 CVAE models are trained on the objective Adaptation with different seeds for initialising the model, illustrating the seed dependency problem with VAEs. Some seeds result in models capable of outputting multiple distributions conditional on the prompt, while others result in models that get stuck and can only output a single kind of distribution irrespective of the prompt.

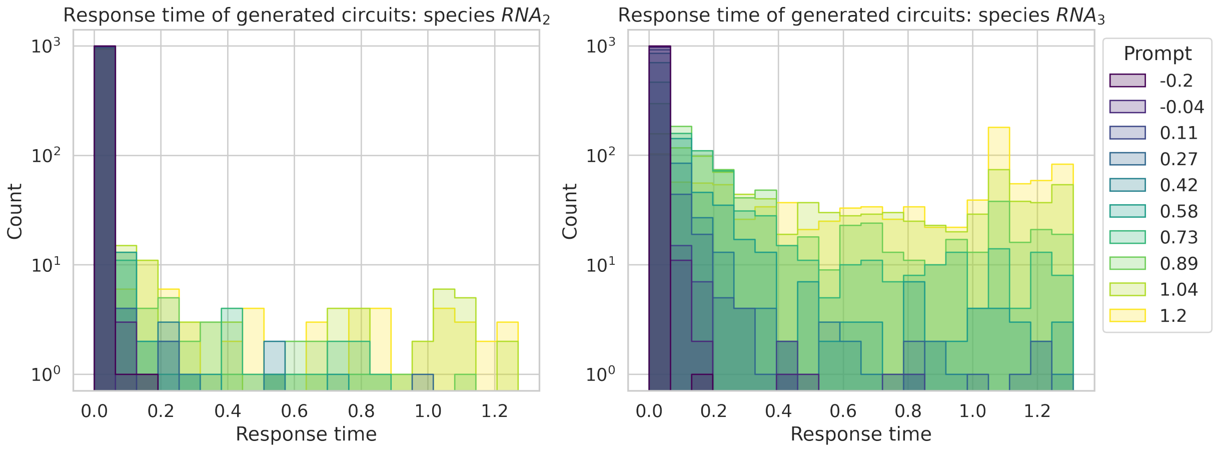

Supplementary Figure 9: Distribution of response times for output RNA_3 (right) and facilitator node RNA_2 (left) of 10,000 circuits generated by a CVAE trained on the objective **response time** prompted at 10 different response times (normalised to $[0, 1]$ for training). Circuits with the highest true response times in RNA_3 (right) are generated by the highest prompt, while prompts at or below 0 are the most specific and generate circuits < 0.2 response time. The facilitator node RNA_2 (left) in some cases also gains a higher response time for the highest prompts to support this function in RNA_3, although only for a fraction of the high response time cases.

Parameter	Value
Seed dataset	1
Objective	Adaptation
Output species	RNA_2
Signal species	RNA_0
Maximal total dataset size	5×10^6
Train split	0.8
Interaction type	Binding energies
Filter x nans	True
Filter c nans	True
Filter sensitivity nans	True
Filter precision nans	True
Filter n same x max	1
Filter n same x max bins	None
Filter out high response time	True
Filter out response times above percent of max	0.8
Transform x with min max	True
Transform c with min max	True
Transform x with robust scaling	True
Transform c with robust scaling	True
Transform x to categorical	False
Transform c to categorical	False
Transform x to categorical one-hot encoding	False
Transform c to categorical one-hot encoding	False
Transform x to negative	True
Transform c to negative	False

Supplementary Table 1: Default hyperparameters – dataset configuration.

Parameter	Value
Seed optimiser	1
Optimiser method	Adam
Optimiser minimum learning rate	1×10^{-6}
Optimiser minimum delta	1×10^{-4}
Learning rate schedule	Cosine decay
Use warm-up	True
Warm-up epochs	20

Supplementary Table 2: Default hyperparameters – training optimiser configuration.

Parameter	Value
Training seed	12
Batch size	256
Epochs	1000
Early-stopping patience	1500
Validation accuracy threshold	0.98
Learning rate	1×10^{-3}
Loss function	Mean square error
Accuracy metric	Accuracy regression
Use dropout	False
Use L2 regularization	False
Use KL divergence	True
KL weight	2×10^{-4}
Use gradient clipping	False

Supplementary Table 3: Default hyperparameters – training loop configuration.

Parameter	Value
Architecture seed	25
Hidden size	32
Latent size encoder	32
Latent size decoder	32
Number of encoder layers	3
Number of decoder layers	3
Model type	CVAE
Use sigmoid activation after decoder	False
Layer initialisation encoder	He Normal
Layer initialisation decoder	He Normal
Initialize model randomly	True
Activation function	Leaky ReLU

Supplementary Table 4: Default hyperparameters – model architecture configuration.
